# Seropositivity to *Campylobacter* and association with abortion and lamb mortality in maiden ewes from Western Australia, South Australia and Victoria

**DOI:** 10.1111/avj.13173

**Published:** 2022-06-05

**Authors:** T Clune, M Bruce, E Glanville, AJD Campbell, A Lockwood, S Hancock, AN Thompson, S Beetson, D Brookes, C Trengove, R O'Handley, C Jacobson

**Affiliations:** ^1^ Centre for Animal Production and Health, Food Futures Institute Murdoch University South Street Murdoch Western Australia 6150 Australia; ^2^ Centre for Biosecurity and One Health, Harry Butler Institute Murdoch University South Street Murdoch Western Australia 6150 Australia; ^3^ Mackinnon Project, Faculty of Veterinary and Agricultural Sciences University of Melbourne Werribee Victoria 3030 Australia; ^4^ Nossal Institute for Global Health, Melbourne School of Population and Global Health University of Melbourne Melbourne Victoria 3010 Australia; ^5^ School of Animal and Veterinary Sciences University of Adelaide Roseworthy South Australia 5371 Australia

**Keywords:** abortion, lamb survival, mortality, ovine campylobacteriosis, primiparous ewe, sheep

## Abstract

This case‐control study investigated associations between *Campylobacter fetus* or *Campylobacter jejuni* titre and reproductive outcomes in 22 flocks of Merino and non‐Merino maiden ewes aged 1–2 years old. *Campylobacter* titres were also determined for multiparous ewes aged 3 years or older on the same farms. *C. fetus* ‘positivity’ (titre ≥1:80) was detected for 12% (57/462; 95% confidence interval [95% CI] 9.6 to 15.6) of maiden ewes and 31% (65/210; 95% CI 25.0 to 37.4) of mature ewes. The odds for failing to rear a lamb in *C. fetus*‐‘exposed’ maiden ewes (titre ≥1:10) was 2.01 times that of seronegative ewes (95% CI 1.09 to 3.77; P = 0.027), but there was no association between *C. fetus‐*‘positivity’ (titre ≥1:80) and failure to rise (OR 1.69; 95% CI 0.77 to 3.76; P = 0.191). *C. fetus* abortions were confirmed with microbial culture in one maiden ewe flock. In this flock, *C. fetus* titres fluctuated and often waned by lamb marking, highlighting the value of necropsies during abortion investigations. *C. jejuni‐*‘positivity’ (titre ≥1:80) was detected for 44% (204/462; 95% CI 39.7 to 48.7) maiden ewes, but odds of failing to rear were decreased for *C. jejuni*‐‘positive’ ewes (OR 0.52; 95% CI 0.32 to 0.83; P = 0.007). The association between *Campylobacter* serology and the reproductive outcome was inconsistent in these flocks. Serology should be considered in the context of other risk factors and used in conjunction with other strategies to investigate the impact of *Campylobacter* exposure on ewe reproductive performance such as monitoring for abortions and lamb necropsies to determine aetiological diagnosis, and vaccination trials.

**Abbreviations**
95% CI: 95% confidence interval
FTR: failure to rear
OR: odds ratio
qPCR: quantitative polymerase chain reaction
SA: South Australia
VIC: Victoria
WA: Western Australia

Infectious agents causing abortion, stillbirths and perinatal lamb mortality can cause significant production losses in sheep flocks. New Zealand studies have reported that infectious agents are an important contributor to the poor and inconsistent reproductive performance observed for maiden ewes.^1^, ^2^, ^3^, ^4^ In Australia, the causes of foetal and lamb losses between pregnancy diagnosis in mid‐pregnancy and lamb marking for maiden ewes are not well studied. Furthermore, it is not clear whether infectious diseases are an important contributor to the poorer reproductive performance reported for maidens compared to multiparous ewes.^5^, ^6^, ^7^, ^8^

Ovine campylobacteriosis caused by *Campylobacter fetus* or *Campylobacter jejuni* is one of the most frequently diagnosed causes of ovine abortion in Australia.^9^, ^10^ Transmission occurs via ingestion of feed and water contaminated by faeces or aborted material, with no evidence of venereal spread in sheep.^11^ Abortion due to campylobacteriosis generally occurs in the last 6 weeks of pregnancy and may be sporadic or associated with an abortion frequency up to 50% of ewes in previously naïve flocks.^12^ The extent of reproductive loss attributable to abortion or perinatal lamb mortality is difficult to quantify, particularly in extensively‐managed sheep flocks. However, it has been estimated that sub‐clinical disease could account for around 10% of foetal and lamb mortality in flocks where *Campylobacter* spp. are endemic.^13^

Ewes that abort due to campylobacteriosis become immune and are less susceptible to abortion or perinatal mortalities with subsequent exposure.^14^, ^15^, ^16^ Younger ewes are less likely to have had previous exposure to *Campylobacter* spp. and are therefore considered at greater risk of abortion due to campylobacteriosis. A commercial vaccine against both *C. fetus* and *C. jejuni* is available in Australia and internationally. Higher lamb marking percentages have been reported from vaccinated compared to unvaccinated maiden ewe flocks in New Zealand.^1^ However, Australian studies report variable responses to *Campylobacter* vaccination in maiden ewes.^3^, ^17^, ^18^ Further investigation is required to quantify the impact of campylobacteriosis on the reproductive performance of maiden ewes in Australia and risk factors for disease.

The incidence of campylobacteriosis in Australian sheep is not well described. A serological survey by the manufacturer of a vaccine against *Campylobacter* spp. reported individual animal seroprevalence of 30% for *C. fetus* (titre ≥1:10) and 41% for *C. jejuni* (titre ≥1:80) for ewes across the major sheep production regions of Australia.^19^ This was consistent with data from veterinary laboratories indicating that *Campylobacter* abortions are diagnosed for sheep located across different states of Australia in most years.^9^, ^10^ However, interpreting the pathological significance of serology results is complicated because *Campylobacter* spp. are commonly isolated from the gastrointestinal tract of clinically healthy sheep.^20^, ^21^, ^22^, ^23^ An improved understanding of *Campylobacter* antibody dynamics in relation to sheep reproductive outcomes will improve our ability to estimate the impacts of *Campylobacter* on the health and productivity of sheep based on serological studies, and support veterinarians in making evidence‐based recommendations on disease management based on serology.

The aims of this study were to: (1) investigate associations between seropositivity to *C. fetus* and *C. jejuni* and reproductive outcomes for maiden ewes, and (2) determine appropriate strategies for estimating the impact of campylobacteriosis on abortion and lamb mortality using ewe serology.

## Materials and methods

All procedures were conducted according to guidelines of the Australian Code of Practice for the Use of Animals for Scientific Purposes and were approved by the Murdoch University Animal Ethics Committee (R3004/17). Consent to participate was provided by the owners of the sheep included in this study.

### Animals, study sites and management

**Figure 1:**
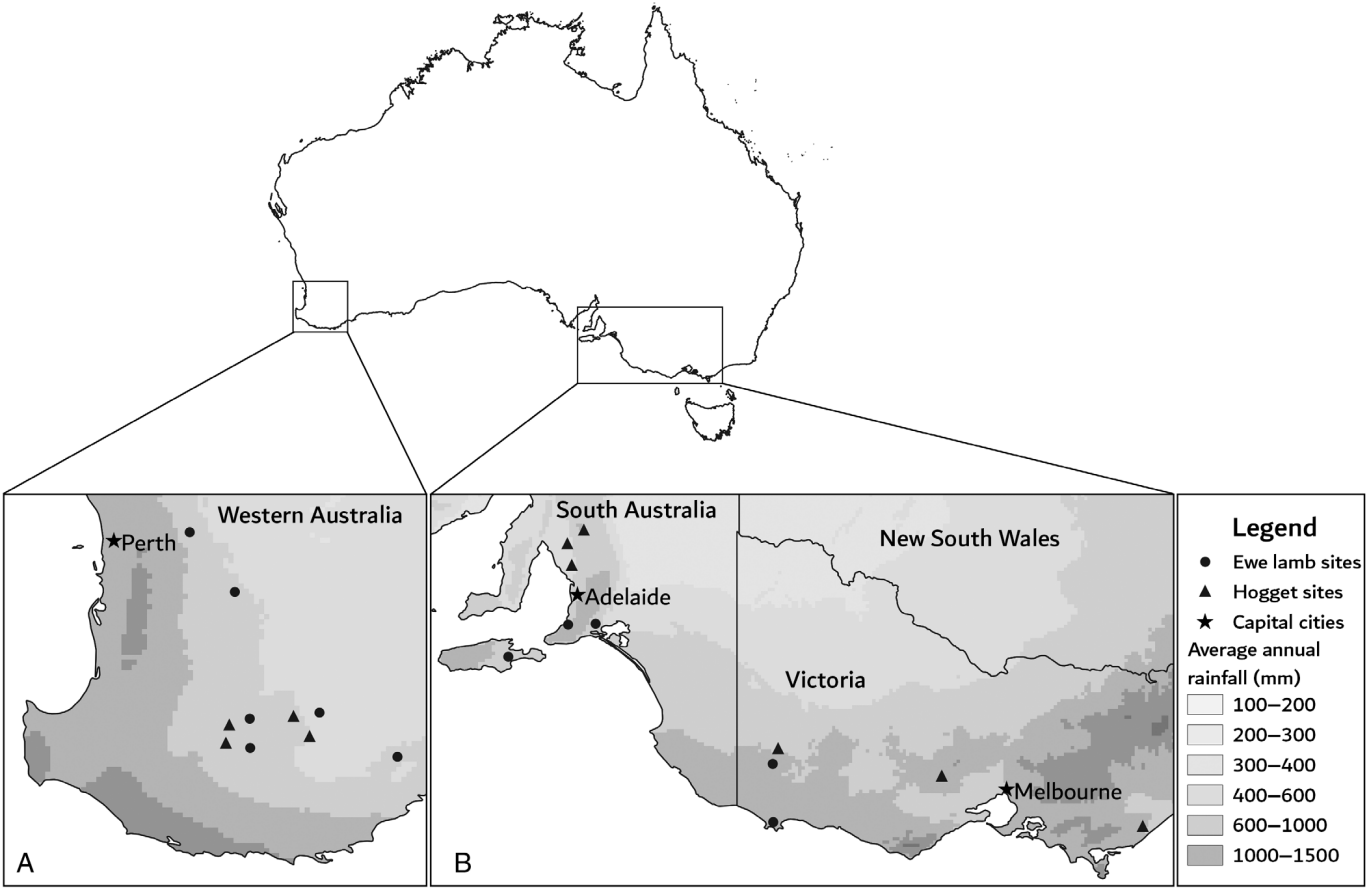
Approximate location of 21 farms where maiden and mature ewes were sampled in Western Australia (A) and South Australia and Victoria (B). On one farm, two flocks were sampled. Average annual rainfall data was sourced from the Australian Government Bureau of Meteorology.^39^

This case‐control study was nested within a larger cohort study, which involved monitoring maiden ewes during pregnancy and lambing as described by Clune et al.^24^ A subset of 22 flocks from 21 farms that had not received *Campylobacter* spp. vaccination was included in this study. Maiden ewes were joined for an average of 39 days, ranging from 17 to 54 days. These flocks were located across a range of geographic regions and rainfall zones across Western Australia (WA) (n = 11), South Australia (SA) (n = 6) and Victoria (VIC) (n = 5; Table 1; Figure 1). Briefly, data (including condition score, liveweight and reproductive outcome) were collected for approximately 200 ewes per flock over a single breeding season between 2018 and 2020. Flock 3 (2018) and flock 14 (2019) were located on the same farm, but all other flocks were on different farms. Farms were selected based on the following inclusion criteria: sufficient maiden ewes (approximately 200 mated), ability to monitor ewes and their progeny over the study period, and sheep genotype and management that were generally representative of standard commercial sheep farms in the region. Some stud flocks were included in the study which may have increased the frequency of monitoring relative to commercial flocks, but stocking rate (density) and housing were broadly comparable to commercial sheep flocks in these regions. Flock reference codes were assigned in order of recruitment for the larger cohort study.^24^ Flocks that had received *Campylobacter* spp. vaccination were subsequently excluded from this study; hence the flock reference codes are not sequential (Table 1).

**Table 1:** Location of farms, historical average annual rainfall, ewe breed, frequency of mid‐pregnancy abortion between scan 1 and scan 2 and overall foetal/lamb mortality between scan 1 and lamb marking for maiden ewe lambs and hoggets in southern Australia between 2018 and 2020

Flock reference	Location	Rainfall (mm/annum)	Breed	Mid‐pregnancy abortion^a^ (% ewes)	Overall foetus and/or lamb mortality^b^ (% foetuses)
Ewe lambs					
3^c^	Narrogin, WA	545	Composite	7.4	37.8^d^
4	York, WA	392	Composite	1.5	23.0
7	Kojonup, WA	530	Composite	0.7	27.7^d^
8	Katanning, WA	444	Merino	1.2	33.0
11	Kojonup WA	530	Dorper	0.0	27.0^d^
14^d^	Narrogin, WA	545	Composite	23.8	59.0^d^
16	Ongerup, WA	387	White Suffolk	2.9	33.8^d^
19	Nareen, VIC	691	Composite	8.5	50.5^d^
20	Cashmore, VIC	841	Composite	4.3	41.1
23	Kangaroo Island, SA	530	Composite	1.8	18.1
25	Sellicks Hill, SA	493	Composite	1.4	65.5
30	Strathalbyn, SA	490	Border Leicester	1.3	37.7
Hoggets					
1	Kojonup, WA	530	Merino	0	19.7^d^
2	Kojonup, WA	530	Merino	0	30.1^d^
5	Korunye, SA	364	Merino	1.1	27.4
9	Watervale, SA	650	Merino	0	21.7
10	Broomehill, WA	446	Merino	0	26.3
12	Tarlee, SA	469	Merino	1.1	28.6
13	Giffard West, VIC	662	Merino	0.9	52.7
15	Katanning, WA	444	Merino	0.5	25.4
26	Culla, VIC	579	Merino	4.4	38.9
29	Ballarat, VIC	686	Merino	2.2	23.3

^a^ Ewes with mid‐pregnancy abortion between scan 1 and scan 2 as the proportion (%) of ewes scanned pregnant at scan 1. Includes all causes of mid‐pregnancy abortion (i.e., not specific to campylobacteriosis).

^b^ Overall foetal and lamb loss between scan 1 and lamb marking expressed as proportion (%) foetuses detected at scan 1. Includes all causes of foetal/lamb mortality (i.e., not specific to campylobacteriosis).

^c^ Same farm – primiparous ewes tested in 2018 (flock 3) and 2019 (flock 14).

^d^ Tissues from aborted or stillborn lambs submitted for *Campylobacter* spp. microbial culture and/or qPCR.

qPCR, quantitative polymerase chain reaction; SA, South Australia; VIC, Victoria; WA, Western Australia.

Maiden ewes were mated as either ewe lambs (7–10 months, n = 12 flocks) or maiden hoggets (18–20 months, n = 10 flocks), with both Merino and non‐Merino ewes included in the study (Tables 1 and 2). Ewes in this study were not vaccinated against *Campylobacter* spp. However, some farms had other cohorts of ewes on the same property that had received Coopers Ovilis® Campyvax® *Campylobacter* vaccine for sheep (Coopers, MSD Animal Health, VIC, Aust). Each farm ran self‐replacing flocks (i.e., ewes were born and raised on the study farm) and maiden ewes were managed extensively as per standard farm practice. At each farm, 10–20 unvaccinated, multiparous ewes aged 3 years or older that had been bred on the farm were randomly selected for blood sampling at a single time‐point during the study period.

**Table 2:** Ewe, flock and *Campylobacter* spp. titre category definitions

Category	Definition
Ewe lambs	Primiparous ewe mated at 7–10 months of age
Maiden hoggets	Primiparous ewe mated at 18–20 months of age
Mature ewes	Multiparous ewes aged 3‐years of age or older
Fail to rear	Maiden ewe determined to be pregnant at scan 1 that subsequently failed to rear a lamb to lamb marking
Exposed ewe	Ewe with *C. fetus/C. jejuni* titre ≥1:10
Exposed flock	Ewe flock (ewe lamb, maiden hogget or mature ewe flock) with *C. fetus/C. jejuni* titre ≥1:10 detected in at least one ewe
Exposed farm	Farm with *C. fetus/C. jejuni* titre ≥1:10 detected in at least one ewe (regardless of age)
Positive ewe	Ewe with *C. fetus/C. jejuni* titre ≥1:80
Positive flock	Ewe flock (ewe lamb, maiden hogget or mature ewe flock) with *C. fetus/C. jejuni* titre ≥1:80 detected in at least one ewe
Positive farm	Farm with *C. fetus/C. jejuni* titre ≥1:80 detected in at least one ewe (regardless of age)

### Determination of reproductive outcome

The reproductive outcome for maiden ewes was determined using two sequential transabdominal pregnancy ultrasounds (scans) plus observations at lambing rounds and lamb marking as previously described by Clune et al.^24^ Briefly, pregnancy scans were conducted at approximately 85 days (range 62–101; scan 1) and 118 days (range 107–136; scan 2) from the start of mating. Pregnancy scanning for foetal number and viability was performed by experienced researchers, veterinarians or private contractors. The birth type (single, twin or triplet) and survival status (dead or alive) for lambs were recorded within 24 h of birth. Lamb survival and ewe lactation status (lactating or not) were recorded at lamb marking approximately 6 weeks from the start of lambing.

Lamb mortality was calculated based on the number of foetuses identified at scan 1 and the number of lambs marked. Mortalities were classified as ‘mid‐pregnancy abortion’ based on evidence of pregnancy loss between scan 1 and scan 2, plus validation with lambing records (no lamb allocated to ewe at lambing inspections) and ewe lactation status (ewe not lactating at lamb marking). During pregnancy, ewes were inspected by farm staff at least twice weekly by observing the ewes in their paddocks. This included observation for evidence of breech staining, foetal membranes or aborted/premature lambs. For flocks where mid‐pregnancy abortion was detected at scan 2, farm staff were alerted to the possibility of detecting aborted foetuses and the ewes were subsequently checked at least every second day. Ewes that were pregnant at scan 1 but did not have a lamb survive to marking were categorised as ‘failed to rear’ (Table 2). ‘Failed to rear’ included ewes that aborted or had lambs die during the perinatal period as determined by repeat ultrasound, lambing round records, lamb marking records (no live lamb allocated to ewe present at marking) and ewe lactation status at lamb marking (ewe not lactating).

### Blood sample collection and lamb necropsies

Blood samples were collected for maiden and mature ewes as previously described.^25^, ^26^ Briefly, blood samples were collected for all maiden ewes at five time‐points: pre‐mating, scan 1, scan 2, pre‐lambing (approximately 140 days from the start of mating) and lamb marking. Blood samples for mature ewes (age 3 years or older) were collected at a single time‐point during the study period. Reproductive status, timing of sampling relative to lambing, reproductive outcome and reproductive history were not recorded for mature ewes. Blood samples were not collected for mature ewes at farm 20 because unvaccinated mature ewes were not available. All blood samples were obtained by jugular venepuncture into serum vacutainer tubes with a clot activator. Samples were stored on ice or at 2°C before being centrifuged at 4000 rpm for 10 min. Serum was decanted into 2 mL storage tubes and stored at −20°C prior to serological testing.

If abortions were observed, the aborted foetus and/or foetal membranes were collected for necropsy. Lambs that died during the lambing period were collected for necropsy from a subset of flocks from WA (flocks 1, 2, 3, 7, 11, 14, 16) as previously described by Clune et al.^27^

### Sample selection for case‐control study

The sample size needed for the case‐control study to detect an odds ratio (OR) of 2 was 220 ewes in each group assuming 10% of control ewes had a *C. fetus* titre ≥1:80 at 95% confidence level and 80% power. As 22 farms were included, this sample size was achieved with 10 maiden ewe case‐control pairs per farm.

A subsample of maiden ewes that raised lambs (n = 10 ewes) and failed to raise lambs (n ≥ 10 ewes) were selected for serological testing for each flock (Additional files 1 and 2). Serum samples obtained at lamb marking were used for serology except where samples at marking were not available because the ewe was removed from the study flock by the farmer after abortion was detected. In these cases, samples collected at the latest available timepoint after abortion was detected were used for serology (i.e., serum sample collected at scan 2 or pre‐lambing).

For the flock with *C. fetus* abortions confirmed by microbial culture (flock 19), *C. fetus* serology was also conducted for samples collected at previous timepoints for maiden ewes which had *C. fetus* titres ≥1:10 at the latest available timepoint (Additional file 3).

### Serology

Serological testing was performed by ACE Laboratory Services, Bendigo, VIC, Australia. Antibody titres for *C. fetus* and *C. jejuni* were determined using an Agar Gel Immunodiffusion test. Titres ≥1:10 were categorised as ‘exposed’ and ≥ 1:80 were categorised as ‘positive’ as previously described.^17^, ^18^, ^28^

### Campylobacter *spp. detection in tissues from aborted and stillborn lambs*

Aborted (n = 2) and stillborn (n = 33) lambs were recovered from a subset of seven maiden ewe flocks (flocks 1, 2, 3, 7, 11, 14, 16) in WA (Table 1). Tissue samples were submitted to the Department of Primary Industry and Regional Development Diagnostic Laboratory Services, Perth, WA and screened for *Campylobacter* spp. using quantitative polymerase chain reaction (qPCR) and microbial culture methods as previously reported.^27^ Three aborted foetuses were opportunistically recovered from one flock in VIC (flock 19) and submitted to the Veterinary Diagnostic Services Laboratory, VIC (Department of Jobs, Precincts and Regions, Bundoora, VIC, Aust).

### Statistical analyses

Lamb mortality was calculated for each flock based on the number of foetuses identified at scan 1 and the number of lambs marked. Mid‐pregnancy abortion was expressed as a proportion (%) using the number of ewes with pregnancy loss between scan 1 and scan 2 as a proportion of the number of ewes that were confirmed pregnant at scan 1.

Titres ≥1:10 were categorised as ‘exposed’ and ≥ 1:80 were categorised as ‘positive’ (Table 2). A farm or flock was classified as seropositive if at least one ewe had a titre above the specified threshold.

Seropositivity proportion was calculated based on the number of samples with a titre at or above the specified titre cut‐off as a proportion (%) of the samples tested. Seropositivity proportions were compared using a Pearson Chi‐squared test (two‐tailed). The seropositivity 95% confidence interval (CI) was determined using Jeffrey's method.^29^ The correlation between seropositivity in maiden ewes and adult ewes was determined using bivariate Pearson correlation (two‐tailed). For flock 19, where serology was conducted for samples collected at multiple timepoints, titre for pre‐joining and marking sample timepoints were compared using Wilcoxon matched pair‐signed rank test (two‐tailed).

ORs for failing to raise a lamb were calculated for (1) ‘exposed’ maiden ewes compared to ewes that were not exposed (titre <1:10), and (2) ‘positive’ maiden ewes compared to non‐positive ewes (titre <1:80) using logistic regression with flock included as a fixed effect. ORs were determined for failing to rear for different *Campylobacter* spp. titre categories (namely: (1) 1:10 to 1:40; (2) 1:80; and (3) ≥1:160) compared to titres <1:10 using logistic regression with flock included as a fixed effect. Logistic regression was performed using a generalised linear model (binomial family) with P‐values calculated from the Wald statistic. Maiden ewe age category and state were not included as a fixed‐factors because these were co‐linear with a farm (i.e., only one age group or state per farm).

## Results

### Abortion and lamb mortality for maiden ewe study flocks

Reproductive outcomes for each flock in the larger cohort study are described in more detail by Clune et al.^24^ For the subset of flocks included in this study, the overall foetal and lamb mortality in maiden ewes (i.e., all causes of mortality between scan 1 and marking) ranged from 18% to 66% for ewe lambs and 20%–53% for hoggets (Table 1). Mid‐pregnancy abortion was detected in 11/12 ewe lamb flocks and 6/10 hogget flocks (Table 1). Mid‐pregnancy abortion was detected for 5.7% of ewe lambs that were pregnant at scan 1 (220/4351). The frequency of mid‐pregnancy abortion for ewe lamb flocks ranged from 0% to 23.8% (Table 1). For hogget flocks, mid‐pregnancy abortion was detected in 1.5% of pregnant ewes (16/1886), with the frequency ranging from 0% to 4.4% (Table 1).

### Campylobacter fetus *seropositivity in maiden and mature ewe flocks*

*C. fetus* titres ranged between zero (below detectible limit) and 1:640 in both maiden and mature ewes. Titres ranged between 0 and 1:80 for ewes in most (18/22) maiden flocks, with titres ≥1:160 detected in three flocks from VIC (flocks 19, 20, 26) and one flock from SA (flock 30). No maiden ewes with titres ≥1:10 were detected in four flocks from WA (flocks 3, 7, 8 and 14). However, all farms were ‘exposed’ to *C. fetus* (at least one maiden or mature with titre ≥1:10) and 12/21 (66%) farms were ‘positive’ (at least one ewe with titre ≥1:80; Additional file 1).

There was a trend to a higher proportion of *C. fetus* ‘positive’ flocks (at least one ewe in respective age category with titre ≥1:80) for mature ewes (13/20 flocks, 65%) compared to maiden ewes (8/22 flocks, 36%, P = 0.061, Additional file 1). There was no difference in the proportion of ‘positive’ ewe lamb flocks (4/12 flocks, 33%) compared to maiden hogget flocks (4/10 flocks, 40%; P = 0.774).

The proportion of sampled maiden ewes that were *C. fetus* ‘exposed’ and ‘positive’ is shown in Table 3. Within maiden ewe flocks, up to 100% of sampled ewes were *C. fetus* ‘exposed’ (Additional file 1). The proportion of ‘positive’ ewes ranged from 4.8% to 80% for the 8/22 flocks that had at least one ‘positive’ ewe. The proportion of ewes ‘exposed’ or ‘positive’ to *C. fetus* was higher for mature ewes compared to maiden ewes at both titre thresholds (Table 3). There was no difference in the proportion of ‘positive’ ewes for ewe lambs compared to maiden hoggets (P = 0.163). There were trends towards weak positive correlations between the proportion of *C. fetus* ‘exposed’ (r = 0.381, P = 0.088) or ‘positive’ maiden ewes (r = 0.42, P = 0.057) compared to mature ewes on the same farm, noting that maiden ewes were selected based on case‐control sampling and mature ewes were randomly selected.

**Table 3:** Individual animal seroprevalence with 95% confidence interval (CI) for *C. fetus* and *C. jejuni* in maiden ewe lambs or hoggets selected based on reproductive outcome (raised lambs or failed to rear), and randomly selected mature ewes (*C. fetus* only) across all farms

		Exposed (titre ≥1:10)			Positive (titre ≥1:80)		
	Tested (n)	n	%	95% CI	n	%	95% CI
*C. fetus*							
Maiden ewe lambs	260	84	32.3^a^	26.8 to 38.2	37	14.2^a^	10.4 to 18.9
Maiden hoggets	202	47	23.3^b^	17.8 to 29.4	20	9.9^a^	6.3 to 14.6
Mature ewes	210	114	54.3^c^	47.5 to 60.9	65	31.0^b^	25.0 to 37.4
Total	672	245	36.5	32.9 to 40.2	122	18.2	15.4 to 21.2
*C. jejuni*							
Maiden ewe lambs	260	248	95.4^a^	92.3 to 97.4	121	46.5^a^	40.5 to 52.6
Maiden hoggets	202	195	96.5^a^	93.3 to 98.4	83	41.1^a^	34.5 to 48.0
Total	462	443	95.9	93.8 to 97.4	204	44.2	39.7 to 48.7

Values within *Campylobacter* species and titre category with different superscript letters are significantly different using two sample z‐test to compare sample proportions (two‐tailed) P < 0.05.

### *Association between seropositivity to* C. fetus *and reproductive outcome*

‘Exposed’ maiden ewes had 2.0 higher odds of failing to rear than ewes with no evidence of exposure when adjusted for farm effects (P = 0.027; Table 4 and Additional file 4). In maiden ewes that failed to rear a lamb, an extra 8.1% of ewes were ‘exposed’ to *C. fetus* compared to maiden ewes that raised lambs (32.2% vs. 24.1%; P = 0.054; Additional file 5).

**Table 4:** Odds ratio (OR) and 95% confidence intervals (CI) for failing to rear a lamb (FTR) in ewe lambs and maiden hoggets that were ‘exposed’ (titre ≥1:10) or ‘positive’ (titre ≥1:80) for *C. fetus* and *C. jejuni* compared to ewes with titres below the respective thresholds determined using logistic regression with flock included as fixed effect

			Failure to rear lamb			
	Age category	Exposed^a^ (N)	FTR (n)	OR	95% CI	P‐value
*C. fetus*						
Exposed (titre ≥1:10)	Maiden ewe lambs	84	51	2.16	0.91 to 5.38	0.086
	Maiden hoggets	47	27	1.44	0.75 to 2.81	0.278
	Overall	131	78	2.01	1.09 to 3.77	0.027
Positive (titre ≥1:80)	Maiden ewe lambs	37	26	2.59	1.03 to 6.78	0.047
	Maiden hoggets	20	9	0.55	0.10 to 2.47	0.440
	Overall	57	35	1.69	0.77 to 3.76	0.191
*C. jejuni*						
Exposed (titre ≥1: 10)	Maiden ewe lambs	249	132	0.42	0.08 to 1.62	0.232
	Maiden hoggets	195	99	1.39	0.29 to 7.53	0.679
	Overall	444	231	0.71	0.24 to 1.93	0.506
Positive (titre ≥1:80)	Maiden ewe lambs	121	58	0.46	0.24 to 0.87	0.018
	Maiden hoggets	83	38	0.69	0.29 to 1.22	0.160
	Overall	204	96	0.52	0.32 to 0.83	0.007

^a^ Exposed: Exposed to disease risk (e.g., titre ≥1:10 or ≥ 1:80 as indicated).

There was no evidence of increased odds for failing to rear for ‘positive’ maiden ewes compared to ewes with *C. fetus* titre <1:80 (P = 0.191; Table 4 and Additional file 4). For the subset of ewe lamb flocks, ‘positive’ ewe lambs had 2.59 higher odds of failing to rear a lamb compared to ewe lambs with a titre <1:80 (P = 0.047; Table 4) and an extra 9.4% ewes were ‘positive’ for ewe lambs that failed to rear compared to those that reared lambs (18.6% vs. 9.2%; P = 0.03, Additional file 5).

Across the farms, there was considerable variation in the proportion of ‘exposed’ and ‘positive’ ewes in failed to the rear and reared groups (Additional file 5). Flock 19 (where *C. fetus* abortions were confirmed by culture) was the only flock with a significantly higher proportion of *C. fetus* ‘positive’ ewes for those that failed to rear compared to ewes that raised lambs (85% vs. 20%, P < 0.001; Additional file 5). Eight of the 22 maiden flocks had at least one ‘positive’ ewe. In this subset of eight flocks, there was no significant increase in odds of failing to rear for ‘positive’ ewes compared to ewes with *C. fetus* titre <1:80 (OR: 1.69, 95% CI 0.77 to 3.76, P = 0.191 adjusted for flock). However, this should be interpreted with caution due to low statistical power.

ORs for failing to rear a lamb in ewes with evidence of seropositivity to *C. fetus* at different titre cut‐offs compared to ewes with a titre of <1:10 are shown in Table 5. There was no significant increase in the ORs for failing to rear a lamb at the four *C. fetus* titre cut‐off levels tested compared to ewes with a titre <1:10 (Table 5).

**Table 5:** Odds ratio (OR) and 95% confidence intervals (CI) for failing to rear a lamb (FTR) in maiden ewes variable *C. fetus* and *C. jejuni* titre cut‐off levels determined using logistic regression with flock included as fixed effect

			Failure to rear lamb			
	Titre cut‐off category	Total tested (n)	FTR (n)	OR	95% CI	*P*‐value
*C. fetus*	Titre <1:10	331	164	Reference		
	Titre 1:10 to 1:40^a^	74	31	1.41	0.85 to 2.37	0.184
	Titre 1:80^b^	29	12	1.44	0.67 to 3.19	0.351
	Titre ≥1:160	28	10	1.83	0.84 to 4.24	0.139
*C. jejuni*	Titre <1:10	18	11	Reference		
	Titre 1:10 to 1:40^a^	240	105	1.22	0.47 to 3.42	0.689
	Titre 1:80^b^	136	74	1.88	0.70 to 5.37	0.220
	Titre ≥1:160	68	34	1.57	0.55 to 4.73	0.403

^a^ ‘Exposed’.

^b^ ‘Positive’.

### *Detection of* Campylobacter *spp. in tissues from aborted and stillborn lambs*

*C. fetus* was cultured from liver, lung and abomasal content from three aborted foetuses opportunistically recovered between scan 2 and pre‐lambing from one maiden ewe lamb flock in VIC (flock 19). Dam pedigree was not able to be determined for these aborted foetuses.

Neither *C. fetus* nor *C. jejuni* was detected by qPCR or isolated via culture from samples of aborted or stillborn lambs (n = 35) recovered from a subset of seven flocks in WA.^27^ *Campylobacter sputorum* and *Campylobacter mucosalis* were detected by qPCR and sequencing in placental samples collected from one farm. These were not detected on microbial cultures and are not considered reproductive pathogens.

### *Serial* C. fetus *titres in flock 19*

*C. fetus* abortions were confirmed in flock 19 based on microbial cultures performed on aborted foetuses recovered from ewe lambs. Titres from these ewes fluctuated over time (Additional file 3). There was a significant increase in titre between mating and lamb marking in the ewes that failed to rear (Wilcoxon signed‐rank test, P < 0.001), but not in the ewes that raised lambs (P = 0.72). For the ewe lambs that failed to rear, a *C. fetus* titre ≥1:320 was detected in 4/20 (20%, 95% CI 7.2 to 40.8) ewes at scan 2, 6/10 (60%, 95% CI 30.4 to 84.7) ewes pre‐lambing and 2/20 (10%, 95% CI 2.1 to 28.4) ewes at marking. Titres fell to <1:320 by marking for 6/8 (75%, 95% CI 40.8 to 94.4) ewes that had previously had a *C. fetus* titre ≥1:320 at scan 2 or pre‐lambing (Additional file 3).

### *Seropositivity to* C. jejuni *in maiden ewe flocks*

*C. jejuni* ‘exposure’ was detected on 21/21 (100%) farms and ‘positive’ ewes were detected on 18/21 (86%) farms (Additional file 2). There was no difference in the proportion of ‘positive’ flocks between ewe lambs (9/12 flocks) and hoggets (9/10 flocks; P = 0.368).

The proportion of individual ewes ‘exposed’ and ‘positive’ for *C. jejuni* are shown in Table 3. There was no difference in the proportion of *C. jejuni* ‘positive’ ewe lambs compared to hoggets (P = 0.555). Within maiden ewe flocks, 80%–100% ewes were categorised as ‘exposed’ and 0%–100% ewes were categorised as ‘positive’ for *C. jejuni* (Additional file 2).

### *Association between seropositivity to* C. jejuni *and reproductive outcome*

There was no increase in the odds of failing to rear a lamb in *C. jejuni* ‘exposed’ maiden ewes (P = 0.506; Table 4) and there was no difference in the proportion of *C. jejuni* ‘exposed’ ewes that failed to rear a lamb compared to those that raised lambs (95.0% vs. 96.8%; P = 0.332).

Maiden ewes, and specifically maiden ewe lambs, that were *C. jejuni* ‘positive’ had lower odds of failing to rear a lamb compared to ewes with *C. jejuni* titre <1:80 (Table 4 and Additional file 6). In maiden ewes that failed to rear lambs, 9.4% lesser ewes were ‘positive’ compared to ewes that raised lambs (39.7% vs. 49.1%; P = 0.042). There was no evidence of increased odds of failure to rear for ewes with *C. jejuni* titres (1) 1:10–1:40, (2) 1:80, and (3) ≥1:160 compared to titre <1:10 (Table 5).

## Discussion

‘Exposure’ to *Campylobacter* spp. was widespread across the flocks in this study. Maiden ewes that were exposed to *C. fetus* were twice as likely to fail to rear compared to ewes with no evidence of exposure. ‘Positive’ *C. fetus* titres were inconsistently associated with failure to rear and *C. fetus* titre was a poor predictor of failure to rear for the flocks in this study. However, this was confounded by the relatively infrequent detection of titres for *C. fetus* in maiden flocks with no *C. fetus‐*‘exposed’ maiden ewes detected in four flocks and no ‘positive’ maiden ewes detected in 14 flocks. Additionally, there were insufficient ewes with high titres (≥1:160) in this study to determine whether high titres at lamb marking were associated with increased abortion or lamb mortality rates. *C. fetus‐*associated abortion occurred on one farm, consistent with the previously reported sporadic nature of campylobacteriosis in Australian flocks.^9^ Whilst *C. jejuni* was detected in all flocks, there was no evidence that seropositivity to *C. jejuni* was associated with increased odds of failing to rear at either titre threshold.

It is common practice in Australia to screen flocks with disappointing lamb marking rates for seropositivity to *Campylobacter* spp. using the serological test used in this study.^18^ A *C. fetus* titre cut‐off ≥1:80 has been used to indicate a flock as ‘positive’.^18^, ^19^ In our study, *C. fetus* seropositivity based on this cut‐off was associated with higher odds of failing to rear but only in ewe lambs. ‘Exposed’ ewes were detected in flocks that had no evidence of campylobacteriosis abortion or stillbirths based on monitoring ewes and necropsy of aborted and stillborn lambs.^30^ However, lamb necropsies and testing for infectious agents were only performed on a subset of farms. Detection of ‘exposure’ in flocks without evidence of abortion or campylobacteriosis at lamb necropsy could also reflect the persistence of antibodies, infections outside of the period of risk for reproductive disease, insufficient intensity of the infectious challenge, and variations in ewe immunity and strain pathogenicity.^17^, ^31^, ^32^ Alternative strategies for investigating the impact of *C. fetus* exposure on flock reproductive performance could include monitoring ewes for evidence of abortion,^24^ lamb necropsies to determine aetiological diagnoses^9^ and/or a vaccination trial.^13^

*C. fetus* titres ≥1:320 may be associated with campylobacteriosis during abortion storms; however, there were insufficient ewes in this study with titres this high at lamb marking to confidently determine an association with failure to rear. Apart from flock 19 (where campylobacteriosis abortion was confirmed with cultures), ewes with *C. fetus* titre ≥1:320 were only detected in flock 20 (4.3% ewes with mid‐pregnancy abortion) and flock 26 (4.4% ewes with mid‐pregnancy abortion). However, aborted foetuses were not recovered from either of these flocks and lamb necropsies were not performed. Further investigation of antibody dynamics in flocks with campylobacteriosis abortions would be required to determine the positive and negative predictive value of titre ≥1:320. Such investigations should also include lamb necropsies to determine the contribution of infectious agents, including *Campylobacter* spp., to perinatal lamb mortality.

*C. fetus* titres fluctuated during pregnancy for ewes in the one flock with confirmed campylobacteriosis abortion. Titres had declined by marking in many ewes with titre ≤1:160 in 6/8 ewes that had *C. fetus* titre ≥1:320 at scan 2 or pre‐lambing. This indicates that *Campylobacter* spp. serology for a single timepoint at lamb marking or later can result in apparent ‘false negatives’ (i.e., low or moderate titres in flocks where campylobacteriosis abortions occurred in mid‐late pregnancy). Where possible, a suspected diagnosis of campylobacteriosis abortion and perinatal mortality should be based on the detection of *Campylobacte*r spp. at necropsy of the foetus or lamb and not on serology from a single timepoint alone.^28^ In cases where lamb necropsy is not possible, rising titres based on paired samples may be useful in supporting a presumptive diagnosis of campylobacteriosis. However, the relatively rapid change in titres observed in flock 19 indicates that there is a short window of time during an outbreak for the collection of serum samples that will demonstrate this rise.

Immunological naivety is a risk factor for campylobacteriosis abortion with previous studies indicating that convalescent ewes develop protective immunity.^14^, ^33^ This was consistent with our observations in flock 19 where 3/10 ewes that raised lambs had *C. fetus* titre 1:160 at mating, and only 3/20 sampled ewes that subsequently failed to rear lambs were determined to be ‘exposed’ to *C. fetus* at mating (Additional file 3). Foetal or lamb mortality in the three ewes with serological evidence of exposure to *C. fetus* prior to mating could reflect other causes of abortion and perinatal mortality acting simultaneously within the same flock.^9^ An alternate explanation is that prior *C. fetus* exposure was not sufficient to develop protective immunity in these ewes.

This study was not designed as a seroprevalence survey. Nonetheless, detection of seropositivity to *C. fetus* and *C. jejuni* on sheep farms and farm‐ and animal‐level seroprevalence observed in our study were consistent with previous serological ‘surveys’ suggesting that exposure is common on Australian sheep farms. However, those surveys also preferentially sampled ewes that had failed to rear lambs.^18^, ^19^ Evidence of widespread exposure to *Campylobacte*r spp. for Australian sheep located over a wide geographical region was consistent with recent reviews of Australian abortion investigations that showed *Campylobacter* spp. abortions were diagnosed across southern Australian states in most years.^9^, ^10^ Serological evidence of ‘exposure’ to *C. jejuni* in this study was consistent with other studies reporting that *C. jejuni* is commonly detected in Australian sheep without evidence of disease.^21^, ^22^ Notwithstanding the difference in selection criteria for maiden ewes (case‐control) and mature ewes (random selection), *C. fetus* seroprevalence was higher for mature ewes compared to maiden ewes. This likely reflects cumulative age‐related exposure as older ewes have had more time to be exposed to infection, and potentially develop immunity.

An important limitation of serological surveys is that seropositivity does not provide information on the current infection status or causality of foetal or lamb mortality. This study focussed on *Campylobacter* spp.; however, there are other important infectious and non‐infectious causes of abortion and lamb mortality that are often multifactorial.^34^ Necropsies performed on a subset of farms in this study identified dystocia, stillbirth and starvation‐mismothering as cause‐of‐death for the majority of perinatal mortalities based on gross pathology.^27^ This was consistent with other Australian studies reporting the cause of death in lambs.^35^, ^36^ Apart from *Campylobacter* spp., other endemic diseases were identified in some flocks in this study. Abortions, stillbirths and polyarthritis associated with *Chlamydia pecorum* were identified in a subset of farms from WA.^27^, ^30^ Exposure of ewes to *Toxoplasma gondii*,^37^ *Neospora caninum* ^25^ and *Coxiella burnetii* ^26^ were identified on farms in this study, but there was no evidence that these were important contributors to foetal and lamb mortality in these flocks. Further investigations using data from this study will include multivariable analysis to evaluate the relative importance of different pathogens on reproductive performance. Prioritisation and implementation of preventative measures for campylobacteriosis should be considered in the context of the multiple aetiologies for foetal and lamb mortality in maiden ewes, including farm and flock level risk factors. Important risk factors for clinical campylobacteriosis include the environment (e.g., high rainfall, short feed) and management (e.g., high stocking rates, confined feeding, open flocks) during pregnancy.^12^, ^38^

There were several other limitations to this study. Serology was determined using Agar Gel Immunodiffusion. This method has been used by other studies,^17^, ^18^ but sensitivity and specificity of the test are poorly defined. Further validation of the test for field investigations would improve the prediction of true incidence of infection with *Campylobacter* spp. Lamb necropsies were only performed for a subset of eight flocks, of which seven were sampled prospectively and one opportunistically after abortions were observed by the farmer. It is possible that foetal and lamb mortality is associated with *Campylobacter* spp. occurred in the other 14 flocks but were not detected due to lack of necropsy. Some farms in this study had sheep studs. Whilst a requirement for inclusion in the study was that sheep were managed extensively at stocking rates broadly comparable to commercial sheep production in the region, risk factors for campylobacteriosis are not well defined. It is possible that differences in the management of sheep between farms impacted the risk for campylobacteriosis, and additional yarding and monitoring of ewes during pregnancy for the project may have impacted the risk of exposure to *Campylobacter* spp. as well as the risk of lamb mortality. Further investigation with a greater number of farms in each state and expanding the number of farms tested in higher rainfall areas would be required to provide a more accurate assessment of *Campylobacter*‐associated abortion and lamb mortality in different farming regions. Further investigation should also consider an assessment of the interaction between environmental factors and stocking rate as risk factors for disease outbreaks. This would inform region‐specific recommendations relating to interpretation for *Campylobacter* spp. serology, strategies for monitoring ewes using serology and expected cost‐benefit of implementing vaccination.

## Conclusion

Seropositivity to *C. fetus* and *C. jejuni* were detected on most farms. Maiden ewes with serological evidence of exposure (titre ≥1:10) to *C. fetus* had twice the odds of failing to rear a lamb than non‐exposed ewes. Higher odds of failing to rear were observed for positive (titre ≥1:80) ewe lambs but not maiden hogget ewes. There was no evidence that *C. jejuni* serology was a useful indicator for the reproductive outcome which likely reflected the widespread distribution and commensal nature of *C. jejuni*. Abortions associated with *C. fetus* were only detected on one farm using lamb necropsy. In this flock, *C. fetus* titres fluctuated during pregnancy and lactation in ewes that both reared and failed‐to‐rear lambs, reinforcing the value of foetal or lamb necropsy to determine an aetiological diagnosis for abortion and perinatal mortality. Campylobacteriosis is associated with reproductive loss in maiden ewes on some farms for some years. On farms with evidence of serological exposure to *C. fetus*, strategies to determine an association with the reproductive disease include monitoring ewes for abortions and determining aetiological diagnoses for foetal and lamb mortality using necropsies or vaccination trials. Further investigation is warranted to inform region‐specific recommendations relating to interpretation of *Campylobacter* spp. serology and preventative measures.

## Conflicts of interest and sources of funding

Susan Beetson is an Editorial Board member of the Australian Veterinary Journal and co‐author of this article. Dr Beetson was excluded from the peer‐review process and all editorial decisions related to the acceptance and publication of this article. Peer review was handled independently by members of the Editorial Board to minimise bias. The authors have no conflicts of interest to declare. This project was funded by Meat and Livestock Australia (B.AHE.0318). Meat and Livestock Australia approved the study design and approved the manuscript for publication but was not involved in the collection, analysis or interpretation of data, or in the writing of the manuscript. Molecular diagnostic testing for aborted and stillborn lambs was performed under the Western Australian Ewe Abortion and Newborn Lamb Death Surveillance Program (Department of Primary Industries and Regional Development, Western Australia). Tom Clune received post‐graduate scholarships from Meat and Livestock Australia and Sheep Industry Business Innovation (Department of Primary Industries and Regional Development, Western Australia). Some equipment used for this project was funded by the Murdoch University Veterinary Trust.

## Supporting information


**Table S1**. Flock‐level *Campylobacter fetus* seropositivity in maiden ewe lambs or hoggets based on reproductive outcome and randomly selected mature ewes on the same farms.

**Table S2**. Flock‐level *Campylobacter jejuni* seropositivity in maiden ewes lambs or hoggets based on reproductive outcome.

**Table S3**. Serial *Campylobacter fetus* titres for maiden ewes in flock 19.

**Table S4**. Odds ratios (OR) for failing to rear a lamb in maiden ewe lambs or hoggets above and below different *C. fetus* titre cut‐offs with 95% confidence interval (95% CI) and two‐tailed Fisher's exact test for significance.

**Table S5**. Within‐flock comparison for *Campylobacter fetus* seroprevalence in maiden ewes that failed to rear (FTR) and ewes that reared lambs (rear) with two‐way Pearson Chi‐square test.

**Table S6**. Odds ratios (OR) for failing to rear a lamb in maiden ewe lambs or hoggets above and below different *Campylobacter jejuni* titre cut‐off with 95% confidence interval (95% CI) and two‐tailed Fisher's exact test for significance.

## References

[1] Kenyon PR , Thompson AN , Morris ST . Breeding ewe lambs successfully to improve lifetime performance. Small Rumin Res 2014;118:2–15. 10.1016/j.smallrumres.2013.12.022.

[2] Howe L , Collett MG , Pattison RS et al. Potential involvement of *Neospora caninum* in naturally occurring ovine abortions in New Zealand. Vet Parasitol 2012;185:64–71. 10.1016/j.vetpar.2011.10.033. 22112976

[3] Kenyon PR , Pinchbeck GL , Perkins NR et al. Identifying factors which maximise the lambing performance of hoggets: a cross sectional study. N Z Vet J 2004;52:371–377. 10.1080/00480169.2004.36454. 15768138

[4] Ridler AL , Vallee E , Corner RA et al. Factors associated with fetal losses in ewe lambs on a New Zealand sheep farm. N Z Vet J 2015;63:330–334. 10.1080/00480169.2015.1037813. 25980526

[5] Allworth MB , Wrigley HA , Cowling A . Fetal and lamb losses from pregnancy scanning to lamb marking in commercial sheep flocks in southern New South Wales. Animal Prod Sci 2017;57:2060–2065. 10.1071/AN16166.

[6] Kilgour R . Lambing potential and mortality in merino sheep as ascertained by ultrasonography. Aust J Exp Agric 1992;32:311–313. 10.1071/EA9920311.

[7] Kleemann DO , Walker SK . Fertility in south Australian commercial merino flocks: sources of reproductive wastage. Theriogenology 2005;63:2075–2088. 10.1016/j.theriogenology.2004.06.017. 15826674

[8] Hutchison D , Clarke BE , Hancock S et al. Lower reproductive rate and lamb survival contribute to lower lamb marking rate in maiden ewes compared to multiparous ewes. Animals 2022;12:513. 10.3390/ani12040513. 35203221 PMC8868299

[9] Clune T , Beetson S , Besier S et al. Ovine abortion and stillbirth investigations in Australia. Aust Vet J 2021;99:72–78. 10.1111/avj.13040. 33289077

[10] Refshauge G , Atkinson T , Robertson SM et al. Reducing kid loss – select and project. Phase 1 Final report. North Sydney, New South Wales, Meat and Livestock Australia, 2020 Available at: https://www.mla.com.au/research-and-development/reports/2020/reducing-kid-loss-select-and-protect---phase-1/. Accessed October 2021.

[11] Mearns R . Other infectious causes of abortion. In: Aitken ID , editor. Diseases of sheep. 4th edn. Blackwell Publishing, Oxford, 2007;127–137.

[12] Clough W. *A review of ovine Campylobacter in Australia*. Proceedings of the Australian Sheep Veterinarians Annual Conference. Australian Sheep Veterinarians, Cairns, 2003:181–184.

[13] Anderson P. *The implications of campylobacter infections in ewe flocks*. Proceedings of the Society of Sheep and Beef Cattle Veterinarians of the New Zealand Veterinary Association, 2001.

[14] Jensen R , Miller VA , Hammarlund MA et al. Vibrionic abortion in sheep. I. Transmission and immunity. Am J Vet Res 1957;18:326–329. 13424924

[15] Marsh H , Firehammer BD , Scrivner LH . The negative role of the ewe in the transmission of vibriosis of sheep. Am J Vet Res 1954;15:352–355. 13171492

[16] Meinershagen WA , Frank FW , Hulet CV et al. Immunity in ewes resulting from natural exposure to *Vibrio fetus* . Am J Vet Res 1969;30:203–206. 5392977

[17] Glanville EJ . The effect of vaccination against *Campylobacter* on maiden ewe reproduction in Victoria [thesis]. University of Melbourne, 2017.

[18] Walsh J. *Campylobacter infection in sheep: Field survey results and vaccination outcomes*. Proceedings of the Australian Sheep Veterinarians Annual Conference, Dubbo, 2016:158–163.

[19] Wills F . Survey of Campylobacter fetus and campylobacter jejuni seroprevalence in Australian sheep. Sheep Goats and Camelid Veterinarians (Australian Veterinary Association), Wagga Wagga, 2021.

[20] Bailey GD , Vanselow BA , Hornitzky MA et al. A study of the foodborne pathogens: *Campylobacter*, *listeria* and *Yersinia*, in faeces from slaughter‐age cattle and sheep in Australia. Commun Dis Intell 2003;27:249–257. 10.33321/cdi.2003.27.47 12926738

[21] Yang R , Abraham S , Gardner GE et al. Prevalence and pathogen load of *Campylobacter* spp., *Salmonella enterica* and *Escherichia coli* O157/O145 serogroup in sheep faeces collected at sale yards and in abattoir effluent in Western Australia. Aust Vet J 2017;95:143–148. 10.1111/avj.12572. 28444752

[22] Yang R , Jacobson C , Gardner G et al. Longitudinal prevalence, faecal shedding and molecular characterisation of *Campylobacter* spp. and *Salmonella enterica* in sheep. Vet J 2014;202:250–254. 10.1016/j.tvjl.2014.08.001. 25175721

[23] Milnes AS , Stewart I , Clifton‐Hadley FA et al. Intestinal carriage of verocytotoxigenic *Escherichia coli* O157, *salmonella*, thermophilic *Campylobacte*r and *Yersinia enterocolitica* in cattle, sheep and pigs at slaughter in Great Britain during 2003. Epidemiol Infect 2008;136:739–751. 10.1017/S0950268807009223. 17655782 PMC2870870

[24] Clune T , Lockwood A , Hancock S et al. Abortion and lamb mortality between pregnancy scanning and lamb marking for maiden ewes in southern Australia. Animals 2022;12:10. 10.3390/ani12010010. PMC8749747 35011116

[25] Clune T , Lockwood A , Hancock S et al. *Neospora caninum* is not an important contributor to poor reproductive performance of primiparous ewes from southern Australia: evidence from a cross‐sectional study. Parasitol Res 2021;120:3875–3882. 10.1007/s00436-021-07328-z. 34599357

[26] Clune T , Lockwood A , Hancock S et al. *Coxiella burnetii* seroprevalence in primiparous and multiparous ewes from southern Australia: A cross‐sectional study. Comp Immunol Microbiol Infect Dis 2022;80:101727. 10.1016/j.cimid.2021.101727. 34875542

[27] Clune T , Besier S , Hair S et al. *Chlamydia pecorum* detection in aborted and stillborn lambs from Western Australia. Vet Res 2021;52:84. 10.1186/s13567-021-00950-w. 34116730 PMC8196467

[28] Dempster RP , Wilkins M , Green RS et al. Serological survey of *Toxoplasma gondii* and *Campylobacter fetus* in sheep from New Zealand. N Z Vet J 2011;59:155–159. 10.1080/00480169.2011.579240. 21660843

[29] Brown LD , Cat TT , DasGupta A . Interval estimation for a proportion. Stat Sci 2001;16:101–133.

[30] Ostfeld N , Islam MM , Jelocnik M et al. *Chlamydia pecorum*‐induced arthritis in experimentally‐induced and naturally infected sheep. Vet Pathol 2020;58:346–360. 10.1177/0300985820973461. 33208021

[31] Sahin O , Fitzgerald C , Stroika S et al. Molecular evidence for zoonotic transmission of an emergent, highly pathogenic *Campylobacter jejuni* clone in the United States. J Clin Microbiol 2012;50:680–687. 10.1128/jcm.06167-11. 22189122 PMC3295108

[32] Grogono‐Thomas R , Blaser MJ , Ahmadi M et al. Role of S‐layer protein antigenic diversity in the immune responses of sheep experimentally challenged with *Campylobacter fetus* subsp. fetus. Infect Immun 2003;71:147–154. 10.1128/IAI.71.1.147-154.2003. 12496160 PMC143156

[33] Frank FW , Waldhalm DG , Meinershagen WA et al. Newer knowledge of ovine vibriosis. J Am Vet Med Assoc 1965;147:1313–1318. 4160763

[34] Jacobson C , Bruce M , Kenyon PR et al. A review of dystocia in sheep. Small Rumin Res 2020;192:106209. 10.1016/j.smallrumres.2020.106209.

[35] Hinch G , Brien F . Lamb survival in Australian flocks: a review. Anim Prod Sci 2014;54:656–666.

[36] Bruce M , Young JM , Masters DG et al. The impact of lamb and ewe mortality associated with dystocia on Australian and New Zealand sheep farms: a systematic review, meta‐analysis and bio‐economic model. Prev Vet Med 2021;196:105478. 10.1016/j.prevetmed.2021.105478. 34487918

[37] Clune T, Lockwood A, Hancock S. et al. *Toxoplasma gondii* is not an important contributor to poor reproductive performance of primiparous ewes from southern Australia: a prospective cohort study. *BMC Vet Res* 2022;18:109. 10.1186/s12917-022-03211-w. PMC8933891 35305646

[38] Sahin O , Yaeger M , Wu Z et al. *Campylobacter*‐associated diseases in animals. Ann Rev Animal Biosci 2017;5:21–42. 10.1146/annurev-animal-022516-022826. 27860495

[39] Australian Government Bureau of Meteorology . Australian climate averages ‐ rainfall. Available at: http://www.bom.gov.au/jsp/ncc/climate_averages/rainfall/index.jsp. Retrieved June 2021.

