# Supplementary material for: Seropositivity to Campylobacter and association with abortion and lamb mortality in maiden ewes from Western Australia, South Australia and Victoria
Source: Aust Vet J. 2022 Jun 5;100(8):397–406. doi: 10.1111/avj.13173 (PMC9544749; doi:10.1111/avj.13173)
Supplement: Supplementary file 1 — Table S1. Flock‐level Campylobacter fetus seropositivity in maiden ewe lambs or hoggets based on reproductive outcome and randomly selected mature ewes on the same farms. [file AVJ-100-397-s003.docx]

# Additional File 1: Flock-level *C. fetus* seropositivity in maiden ewe lambs or hoggets based on reproductive outcome and randomly selected mature ewes on the same farms

| **Flock reference** | **Location^a^** | ***Maiden - abortion, fail to rear*** *n* (%) | | |  | ***Maiden - reared all lambs*** *n* (%) | | |  | ***Mature ewes*** *n* (%) | | | |  |
| --- | --- | --- | --- | --- | --- | --- | --- | --- | --- | --- | --- | --- | --- | --- |
|  |  | Tested | Exposed ^b^ | Positive ^c^ |  | Tested | Exposed ^b^ | Positive ^c^ |  | Age | Tested | Exposed ^b^ | Positive ^c^ | |
| **EWE LAMBS** | |  |  |  |  |  |  |  |  |  |  |  |  | |
| 3^d^ | Narrogin, WA^e^ | 20 | 0 (0) | 0 (0) |  | 10 | 0 (0) | 0 (0) |  | 5 years | 10^d^ | 10 (100)^e^ | 9 (90)^e^ | |
| 4 | York, WA | 10 | 1 (10) | 0 (0) |  | 10 | 1 (10) | 0 (0) |  | 5-6 years | 10 | 2 (20) | 1 (10) | |
| 7 ^d^ | Kojonup, WA | 10 | 0 (0) | 0 (0) |  | 10 | 0 (0) | 0 (0) |  | 5-7 years | 10 | 6 (60) | 4 (40) | |
| 8 | Katanning, WA | 10 | 0 (0) | 0 (0) |  | 10 | 0 (0) | 0 (0) |  | 4-6 years | 10 | 4 (40) | 0 (0) | |
| 11 ^d^ | Kojonup WA | 10 | 0 (0) | 0 (0) |  | 10 | 2 (20) | 0 (0) |  | 5-6 years | 10 | 3 (30) | 0 (0) | |
| 14 ^d^ | Narrogin, WA^e^ | 10 | 0 (0) | 0 (0) |  | 10 | 0 (0) | 0 (0) |  | 5 years | 10^d^ | 10 (100)^e^ | 9 (90)^e^ | |
| 16 ^d^ | Ongerup, WA | 10 | 3 (30) | 0 (0) |  | 10 | 2 (20) | 0 (0) |  | 4-7 years | 10 | 1 (10) | 1 (10) | |
| 19 ^d^ | Nareen, VIC | 20 | 20 (100) | 17 (85) |  | 10 | 10 (100) | 2 (20) |  | 5-8 years | 20 | 19 (95) | 15 (75) | |
| 20 | Cashmore, VIC | 10 | 10 (100) | 6 (60) |  | 10 | 8 (80) | 2 (20) |  | 4-8 years | NA^f^ | - | - | |
| 23 | Kangaroo Island, SA | 10 | 6 (60) | 1 (10) |  | 10 | 5 (50) | 4 (40)  (3000000 |  | 4-7 years | 10 | 3 (30) | 2 (20) | |
| 25 | Sellicks Hill, SA | 10 | 8 (80) | 2 (20) |  | 10 | 5 (50) | 3 (30) |  | 4-7 years | 10 | 8 (80) | 2 (20) | |
| 30 | Strathalbyn, SA | 10 | 3 (30) | 0 (0) |  | 10 | 0 (0) | 0 (0) |  | 5+ years | 10 | 9 (90) | 1 (10) | |
| **HOGGETS** | |  |  |  |  |  |  |  |  |  |  |  |  | |
| 1 ^d^ | Kojonup, WA | 10 | 0 (0) | 0 (0) |  | 10 | 1 (10) | 0 (0) |  | 4-7years | 10 | 1 (10) | 0 (0) | |
| 2 ^d^ | Kojonup, WA | 10 | 2 (20) | 0 (0) |  | 10 | 0 (0) | 0 (0) |  | 5 years | 10 | 3 (30) | 0 (0) | |
| 5 | Korunye, SA | 10 | 2 (20) | 0 (0) |  | 10 | 1 (10) | 0 (0) |  | 5+ years | 10 | 6 (60) | 4 (40) | |
| 9 | Watervale, SA | 10 | 3 (30) | 0 (0) |  | 10 | 0 (0) | 0 (0) |  | 5+ years | 10 | 4 (40) | 0 (0) | |
| 10 | Broomehill, WA | 10 | 0 (0) | 0 (0) |  | 10 | 1 (10) | 0 (0) |  | 5-10 years | 10 | 1 (10) | 0 (0) | |
| 12 | Tarlee, SA | 11 | 2 (18) | 0 (0) |  | 10 | 3 (30) | 1 (10) |  | 5+ years | 10 | 5 (50) | 2 (20) | |
| 13 | Giffard West, VIC | 10 | 6 (60) | 0 (0) |  | 10 | 3 (30) | 2 (20) |  | 5 years | 10 | 7 (70) | 6 (60) | |
| 15 | Katanning, WA | 11 | 1 (9) | 0 (0) |  | 10 | 0 (0) | 0 (0) |  | 5-7 years | 10 | 3 (30) | 0 (0) | |
| 26 | Culla, VIC | 10 | 9 (90) | 8 (8) |  | 10 | 10 (100) | 8 (80) |  | 4-9 years | 10 | 9 (90) | 8 (80) | |
| 29 | Ballarat, VIC | 10 | 2 (20) | 1 (10) |  | 10 | 1 (10) | 0 (0) |  | 4-9 years | 10 | 10 (100) | 10 (100) | |

^a^ SA: South Australia, VIC : Victoria, WA : Western Australia

^b^ Exposed = *C. fetus* titre ≥1:10

^c^ Positive = *C. fetus* titre ≥1:80

^d^ Tissues from aborted or stillborn lambs submitted for *Campylobacter* spp. microbial culture and/or qPCR

^e^ Same farm – ewe lamb flocks tested in 2 years, mature ewes tested once only

^f^ NA: Not available (mature ewes vaccinated for *Campylobacter* spp. therefore not tested)
