# Supplementary material for: Seropositivity to Campylobacter and association with abortion and lamb mortality in maiden ewes from Western Australia, South Australia and Victoria
Source: Aust Vet J. 2022 Jun 5;100(8):397–406. doi: 10.1111/avj.13173 (PMC9544749; doi:10.1111/avj.13173)
Supplement: Supplementary file 2 — Table S2. Flock‐level Campylobacter jejuni seropositivity in maiden ewes lambs or hoggets based on reproductive outcome. [file AVJ-100-397-s002.docx]

Additional File 2*:* Flock-level Campylobacter jejuni seropositivity in maiden ewes lambs or hoggets based on reproductive outcome

|  |  | **Maidens - abortion, fail to rear** (*n* (%)) | | |  | **Maidens - reared all lambs** (*n* (%)) | | |
| --- | --- | --- | --- | --- | --- | --- | --- | --- |
| **Farm** | **Location^a^** | Tested | Exposed ^b^ | Positive ^c^ |  | Tested | Exposed ^b^ | Positive ^c^ |
| **EWE LAMBS** |  |  |  |  |  |  |  |  |
| 3 | Narrogin, WA | 20 | 20 (100) | 20 (100) |  | 10 | 10 (100) | 10 (100) |
| 4 | York, WA | 10 | 7 (70) | 6 (60) |  | 10 | 9 (90) | 7 (70) |
| 7 | Kojonup, WA | 10 | 10 (100) | 3 (30) |  | 10 | 10 (100) | 7 (70) |
| 8 | Katanning, WA | 10 | 10 (100) | 6 (60) |  | 10 | 8 (80) | 6 (60) |
| 11 | Kojonup WA | 10 | 10 (100) | 0 (0) |  | 10 | 10 (100) | 0 (0) |
| 14 | Narrogin, WA | 10 | 10 (100) | 8 (80) |  | 10 | 10 (100) | 9 (90) |
| 16 | Ongerup, WA | 10 | 10 (100) | 4 (40) |  | 10 | 10 (100) | 6 (60) |
| 19 | Nareen, VIC | 20 | 16 (80) | 0 (0) |  | 10 | 10 (100) | 0 (0) |
| 20 | Cashmore, VIC | 10 | 10 (100) | 2 (20) |  | 10 | 10 (100) | 6 (60) |
| 23 | Kangaroo Island, SA | 10 | 9 (90) | 3 (30) |  | 10 | 10 (100) | 5 (50) |
| 25 | Sellicks Hill, SA | 10 | 10 (100) | 5 (50) |  | 10 | 10 (100) | 4 (40) |
| 30 | Strathalbyn, SA | 10 | 19 (95) | 1 (10) |  | 10 | 10 (100) | 3 (30) |
| TOTAL |  | 140 | 131 (94) | 58 (41) |  | 120 | 117 (97) | 63 (52) |
|  |  |  |  |  |  |  |  |  |
| **HOGGETS** |  |  |  |  |  |  |  |  |
| 1 | Kojonup, WA | 10 | 10 (100) | 10 (100) |  | 10 | 9 (90) | 8 (80) |
| 2 | Kojonup, WA | 10 | 10 (100) | 10 (100) |  | 10 | 10 (100) | 10 (100) |
| 5 | Korunye, SA | 10 | 10 (100) | 0 (0) |  | 10 | 10 (100) | 2 (20) |
| 9 | Watervale, SA | 10 | 9 (90) | 2 (20) |  | 10 | 10 (100) | 2 (20) |
| 10 | Broomehill, WA | 10 | 10 (100) | 1 (10) |  | 10 | 10 (100) | 4 (40) |
| 12 | Tarlee, SA | 11 | 11 (100) | 5 (45) |  | 10 | 9 (90) | 3 (30) |
| 13 | Giffard West, VIC | 10 | 9 (90) | 0 (0) |  | 10 | 8 (80) | 0 (0) |
| 15 | Katanning, WA | 11 | 11 (100) | 2 (18) |  | 10 | 10 (100) | 6 (60) |
| 26 | Culla, VIC | 10 | 9 (90) | 5 (50) |  | 10 | 10 (100) | 4 (40) |
| 29 | Ballarat, VIC | 10 | 10 (100) | 3 (30) |  | 10 | 10 (100) | 6 (60) |
| TOTAL |  | 102 | 99 (97) | 38 (37) |  | 100 | 96 (96) | 45 (45) |
|  |  |  |  |  |  |  |  |  |
| OVERALL |  | 242 | 230 (95) | 96 (40) |  | 220 | 213 (97) | 108 (49) |

^a^ SA : South Australia VIC : Victoria WA : Western Australia

^b^ Exposed = *C. fetus* titre ≥1:10

^c^ Positive = *C. fetus* titre ≥1:80
