# Supplementary material for: Seropositivity to Campylobacter and association with abortion and lamb mortality in maiden ewes from Western Australia, South Australia and Victoria
Source: Aust Vet J. 2022 Jun 5;100(8):397–406. doi: 10.1111/avj.13173 (PMC9544749; doi:10.1111/avj.13173)
Supplement: Supplementary file 3 — Table S3. Serial Campylobacter fetus titres for maiden ewes in flock 19. [file AVJ-100-397-s005.docx]

# Additional File 3: Serial *Campylobacter fetus* titres for maiden ewes in Flock 19

| **Ewe ID** | **Mating** | **Scan1** | **Scan 2** | **Pre-lambing** | **Marking** |
| --- | --- | --- | --- | --- | --- |
| **Mid-pregnancy abortion (scan 1 – scan 2)** | | |  |  |  |
| 18650 | ≤1:10 | ≤1:10 | 1:320 | 1:320 | 1:160 |
| 18621 | ≤1:10 | ≤1:10 | 1:160 | 1:320 | 1:160 |
| 18698 | ≤1:10 | ≤1:10 | 1:160 | 1:160 | 1:80 |
| 18648 | ≤1:10 | ≤1:10 | 1:80 | 1:160 | 1:80 |
| 18551 | ≤1:10 | ≤1:10 | 1:80 | 1:320 | 1:160 |
| 18654 | ≤1:10 | ≤1:10 | 1:40 | 1:320 | 1:320 |
| 18557 | ≤1:10 | ≤1:10 | 1:40 | 1:320 | 1:40 |
| 18611 | ≤1:10 | ≤1:10 | 1:20 | 1:20 | 1:10 |
| 18595 | ≤1:10 | 1:40 | 1:160 | 1:160 | 1:160 |
| 18538 | 1:20 | 1:160 | 1:320 | 1:640 | 1:80 |
|  |  |  |  |  |  |
| **Fail to rear (late abortion or perinatal lamb death)** | | | |  |  |
| 18546 | <1:10 | NT^A^ | 1:320 | NT | 1:160 |
| 18549 | <1:10 | NT | 1:80 | NT | 1:80 |
| 18548 | <1:10 | NT | 1:80 | NT | 1:80 |
| 18563 | <1:10 | NT | 1:80 | NT | 1:80 |
| 18553 | <1:10 | NT | 1:40 | NT | 1:80 |
| 18643 | <1:10 | NT | 1:40 | NT | 1:80 |
| 18610 | <1:10 | NT | 1:20 | NT | 1:160 |
| 18691 | <1:10 | NT | <1:10 | NT | 1:80 |
| 18571 | 1:80 | NT | 1:320 | NT | 1:320 |
| 18615 | 1:160 | NT | 1:80 | NT | 1:40 |
|  |  |  |  |  |  |
| **Raised lambs** |  |  |  |  |  |
| 18628 | ≤1:10 | NT | NT | NT | 1:160 |
| 18690 | ≤1:10 | NT | NT | NT | 1:80 |
| 18632 | ≤1:10 | NT | NT | NT | 1:40 |
| 18703 | ≤1:10 | NT | NT | NT | 1:20 |
| 18708 | ≤1:10 | NT | NT | NT | 1:20 |
| 18709 | ≤1:10 | NT | NT | NT | 1:20 |
| 18663 | ≤1:10 | NT | NT | NT | 1:10 |
| 18555 | 1:160 | NT | NT | NT | 1:40 |
| 18642 | 1:160 | NT | NT | NT | 1:40 |
| 18625 | 1:160 | NT | NT | NT | 1:10 |

^A^ NT: not tested
