# Supplementary material for: Seropositivity to Campylobacter and association with abortion and lamb mortality in maiden ewes from Western Australia, South Australia and Victoria
Source: Aust Vet J. 2022 Jun 5;100(8):397–406. doi: 10.1111/avj.13173 (PMC9544749; doi:10.1111/avj.13173)
Supplement: Supplementary file 4 — Table S4. Odds ratios (OR) for failing to rear a lamb in maiden ewe lambs or hoggets above and below different C. fetus titre cut‐offs with 95% confidence interval (95% CI) and two‐tailed Fisher's exact test for significance. [file AVJ-100-397-s004.docx]

# Additional File 4: Odds ratios (OR) for failing to rear a lamb in maiden ewe lambs or hoggets above and below different *C. fetus* titre cut-offs with 95% confidence interval (95% CI) and two-tailed Fisher’s exact test for significance

| **Flock reference** | **Location^a^** | ***C. fetus* titre ≥1:10 ^b^** | | |  | ***C. fetus* titre ≥1:80 ^b^** | | |  | ***C. fetus* titre ≥1:160 ^b^** | | |  | ***C. fetus* titre ≥1:320 ^b^** | | |
| --- | --- | --- | --- | --- | --- | --- | --- | --- | --- | --- | --- | --- | --- | --- | --- | --- |
|  |  | OR | 95% CI | P-value |  | OR | 95% CI | P-value |  | OR | 95% CI | P-value |  | OR | 95% CI | P-value |
| **EWE LAMBS** | |  |  |  |  |  |  |  |  |  |  |  |  |  |  |  |
| 3 | Narrogin, WA | - | - | - |  | - | - | - |  | - | - | - |  | - | - | - |
| 4 | York, WA | 1.00 | 0.01, 87.05 | 1.000 |  | - | - | - |  | - | - | - |  | - | - | - |
| 7 | Kojonup, WA | - | - | - |  | - | - | - |  | - | - | - |  | - | - | - |
| 8 | Katanning, WA | - | - | - |  | - | - | - |  | - | - | - |  | - | - | - |
| 11 | Kojonup WA | - | - | 0.474 ^c^ |  | - | - | - |  | - | - | - |  | - | - | - |
| 14 | Narrogin, WA | - | - | - |  | - | - | - |  | - | - | - |  | - | - | - |
| 16 | Ongerup, WA | 1.70 | 0.14, 25.60 | 1.000 |  | - | - | - |  | - | - | - |  | - | - | - |
| 19 | Nareen, VIC | - | - | - |  | 19.38 | 2.42, 278.15 | 0.001 |  | 5.7 | 0.58, 294.8 | 0.203 |  | - | - | 0.54 |
| 20 | Cashmore, VIC | - | - | 0.474 ^c^ |  | 5.43 | 0.60, 79.83 | 0.170 |  | - | - | 0.211 ^c^ |  | - | - | 1.000 ^c^ |
| 23 | Kangaroo Island, SA | 1.47 | 0.19,12.4 | 1.000 |  | 0.18 | 0.00, 2.45 | 0.303 |  | - | - | 0.087 |  | - | - | - |
| 25 | Sellicks Hill, SA | 3.72 | 0.40,53.81 | 0.350 |  | 0.60 | 0.04, 6.94 | 1.000 |  | - | - | - |  | - | - | - |
| 30 | Strathalbyn, SA | - | - | 0.211 ^c^ |  | - | - | - |  | - | - | - |  | - | - | - |
| **HOGGETS** | |  |  |  |  |  |  |  |  |  |  |  |  |  |  |  |
| 1 | Kojonup, WA | - | - | 1.000 ^c^ |  | - | - | - |  | - | - | - |  | - | - | - |
| 2 | Kojonup, WA | - | - | 0.474 ^c^ |  | - | - | - |  | - | - | - |  | - | - | - |
| 5 | Korunye, SA | 2.16 | 0.10,147.09 | 1.000 |  | - | - | - |  | - | - | - |  | - | - | - |
| 9 | Watervale, SA | - | - | 0.211 ^c^ |  | - | - | - |  | - | - | - |  | - | - | - |
| 10 | Broomehill, WA | - | - | 1.000 ^c^ |  | - | - | - |  | - | - | - |  | - | - | - |
| 12 | Tarlee, SA | 0.54 | 0.04, 6.09 | 0.635 |  | - | - | 0.476 ^c^ |  | - | - | - |  | - | - | - |
| 13 | Giffard West, VIC | 3.27 | 0.41, 33.27 | 0.370 |  | - | - | 0.474 ^c^ |  | - | - | - |  | - | - | - |
| 15 | Katanning, WA | - | - | 1.000 ^c^ |  | - | - | - |  | - | - | - |  | - | - | - |
| 26 | Culla, VIC | - | - | 1.000 ^c^ |  | 1.00 | 0.06, 17.08 | 1.000 |  | 2.23 | 0.27, 21.94 | 0.65 |  | 5.49 | 0.41, 327.0 | 0.303 |
| 29 | Ballarat, VIC | 2.16 | 0.10, 147.1 | 1.000 |  | - | - | 1.000 ^c^ |  | - | - | - |  | - | - | - |
| **OVERALL^d^** |  | **2.01** | **1.09, 3.77** | **0.027** |  | **1.69** | **0.77, 3.76** | **0.191** |  | **1.89** | **0.79 ,5.43** | **0.217** |  | **8.75** | **1.29, 179.07** | **0.058** |

^a^ SA: South Australia, VIC : Victoria, WA : Western Australia

^b^ Odds ratio for failure to rear calculated for ewes with specified *C. fetus* titre compared to ewes with titre below specified threshold

^c^ Odds ratio not calculated due to empty cell

^d^ Overall odds ratio calculated using logistic regression (flock included as fixed effect)
