# Supplementary material for: Seropositivity to Campylobacter and association with abortion and lamb mortality in maiden ewes from Western Australia, South Australia and Victoria
Source: Aust Vet J. 2022 Jun 5;100(8):397–406. doi: 10.1111/avj.13173 (PMC9544749; doi:10.1111/avj.13173)
Supplement: Supplementary file 5 — Table S5. Within‐flock comparison for Campylobacter fetus seroprevalence in maiden ewes that failed to rear (FTR) and ewes that reared lambs (rear) with two‐way Pearson Chi‐square test. [file AVJ-100-397-s001.docx]

# 5

| **Flock reference** | **Location^a^** | **Exposed (titre ≥1:10)** | | |  | **Positive (titre ≥1:80)** | | |
| --- | --- | --- | --- | --- | --- | --- | --- | --- |
|  |  | FTR  *n* (%) | Rear  *n* (%) | P-value |  | FTR  *n* (%) | Rear  *n* (%) | P-value |
| **EWE LAMBS** | |  |  |  |  |  |  |  |
| 3^b^ | Narrogin, WA | 0 (0) | 0 (0) | NA |  | 0 (0) | 0 (0) | NA ^e^ |
| 4 | York, WA | 1 (10) | 1 (10) | 1 |  | 0 (0) | 0 (0) | NA |
| 7 ^b^ | Kojonup, WA | 0 (0) | 0 (0) | NA |  | 0 (0) | 0 (0) | NA |
| 8 | Katanning, WA | 0 (0) | 0 (0) | NA |  | 0 (0) | 0 (0) | NA |
| 11 | Kojonup WA | 0 (0) | 2 (20) | 0.136 |  | 0 (0) | 0 (0) | NA |
| 14 ^b^ | Narrogin, WA | 0 (0) | 0 (0) | NA |  | 0 (0) | 0 (0) | NA |
| 16  ^b^ | Ongerup, WA | 3 (30) | 2 (20) | 0.6056 |  | 0 (0) | 0 (0) | NA |
| 19 ^b^ | Nareen, VIC | 20 (100) | 10 (100) | NA |  | 17 (85) | 2 (20) | **0.0005** |
| 20 | Cashmore, VIC | 10 (100) | 8 (80) | 0.136 |  | 6 (60) | 2 (20) | 0.0679 |
| 23 | Kangaroo Island, SA | 6 (60) | 5 (50) | 0.6531 |  | 1 (10) | 4 (40)  (3000000 | 0.1213 |
| 25 | Sellicks Hill, SA | 8 (80) | 5 (50) | 0.1596 |  | 2 (20) | 3 (30) | 0.6056 |
| 30 | Strathalbyn, SA | 3 (30) | 0 (0) | 0.0603 |  | 0 (0) | 0 (0) | NA |
| TOTAL |  | 51 (36) | 33 (28) | 0.126 |  | 26 (19) | 11 (9) | **0.031** |
|  |  |  |  |  |  |  |  |  |
| **HOGGETS** | |  |  |  |  |  |  |  |
| 1 ^b^ | Kojonup, WA | 0 (0) | 1 (10) | 0.3049 |  | 0 (0) | 0 (0) | NA |
| 2 ^b^ | Kojonup, WA | 2 (20) | 0 (0) | 0.136 |  | 0 (0) | 0 (0) | NA |
| 5 | Korunye, SA | 2 (20) | 1 (10) | 0.5312 |  | 0 (0) | 0 (0) | NA |
| 9 | Watervale, SA | 3 (30) | 0 (0) | 0.0603 |  | 0 (0) | 0 (0) | NA |
| 10 | Broomehill, WA | 0 (0) | 1 (10) | 0.3049 |  | 0 (0) | 0 (0) | NA |
| 12 | Tarlee, SA | 2 (18) | 3 (30) | 0.5185 |  | 0 (0) | 1 (10) | 0.3049 |
| 13 | Giffard West, VIC | 6 (60) | 3 (30) | 0.1775 |  | 0 (0) | 2 (20) | 0.136 |
| 15 | Katanning, WA | 1 (9) | 0 (0) | 0.3311 |  | 0 (0) | 0 (0) | NA |
| 26 | Culla, VIC | 9 (90) | 10 (100) | 0.3049 |  | 8 (80) | 8 (80) | 1.000 |
| 29 | Ballarat, VIC | 2 (20) | 1 (10) | 0.5312 |  | 1 (10) | 0 (0) | 0.3049 |
| TOTAL |  | 27 (28) | 20 (20) | 0.241 |  | 9 (9) | 11 (11) | 0.600 |
|  |  |  |  |  |  |  |  |  |
| **OVERALL** |  | 78 (32) | 53 (21) | 0.054 |  | 35 (15) | 22 (10) | 0.142 |

^a^ SA : South Australia, VIC : Victoria, WA : Western Australia

^b^ Tissues from aborted or stillborn lambs submitted for *Campylobacter* spp. microbial culture and/or qPCR
