# Supplementary material for: Seropositivity to Campylobacter and association with abortion and lamb mortality in maiden ewes from Western Australia, South Australia and Victoria
Source: Aust Vet J. 2022 Jun 5;100(8):397–406. doi: 10.1111/avj.13173 (PMC9544749; doi:10.1111/avj.13173)
Supplement: Supplementary file 6 — Table S6. Odds ratios (OR) for failing to rear a lamb in maiden ewe lambs or hoggets above and below different Campylobacter jejuni titre cut‐off with 95% confidence interval (95% CI) and two‐tailed Fisher's exact test for significance. [file AVJ-100-397-s006.docx]

# Additional File 6: Odds ratios (OR) for failing to rear a lamb in maiden ewe lambs or hoggets above and below different *C. jejuni* titre cut-off with 95% confidence interval (95% CI) and two-tailed Fisher’s exact test for significance

| **Flock reference** | **Location^a^** | ***C. jejuni* titre ≥1:10 ^b^** | | |  | ***C. jejuni* titre ≥1:80 ^b^** | | |  | ***C. jejuni* titre ≥1:160 ^b^** | | |  | ***C. jejuni* titre ≥1:320 ^b^** | | |
| --- | --- | --- | --- | --- | --- | --- | --- | --- | --- | --- | --- | --- | --- | --- | --- | --- |
|  |  | OR | 95% CI | P-value |  | OR | 95% CI | P-value |  | OR | 95% CI | P-value |  | OR | 95% CI | P-value |
| **EWE LAMBS** | |  |  |  |  |  |  |  |  |  |  |  |  |  |  |  |
| 3 | Narrogin, WA | - | - | - |  | - | - | - |  | 0.34 | 0.01, 3.85 | 0.633 |  | - | - | - |
| 4 | York, WA | 0.28 | 0.00, 4.35 | 0.582 |  | 0.66 | 0.08, 5.78 | 1.000 |  | 0.46 | 0.05, 3.61 | 0.656 |  | - | - | 1.000 ^c^ |
| 7 | Kojonup, WA | - | - | - |  | 0.20 | 0.02, 1.68 | 0.179 |  | - | - | - |  | - | - | - |
| 8 | Katanning, WA | - | - | 0.474^c^ |  | 1.00 | 0.11, 8.42 | 1.000 |  | - | - | - |  | - | - | - |
| 11 | Kojonup WA | - | - | - |  | - | - | - |  | - | - | - |  | - | - | - |
| 14 | Narrogin, WA | - | - | - |  | 0.46 | 0.01, 10.51 | 1.000 |  | - | - | 1.000 ^c^ |  | - | - | - |
| 16 | Ongerup, WA | - | - | - |  | 0.46 | 0.05, 3.61 | 0.656 |  | - | - | - |  | - | - | - |
| 19 | Nareen, VIC | - | - | 0.272 ^c^ |  | - | - | - |  | - | - | - |  | - | - | - |
| 20 | Cashmore, VIC | - | - | - |  | 0.18 | 0.01, 1.66 | 0.170 |  | - | - | 1.000 ^c^ |  | - | - | - |
| 23 | Kangaroo Island, SA | - | - | 1.000 ^c^ |  | 0.45 | 0.05, 3.67 | 0.650 |  | - | - | - |  | - | - | - |
| 25 | Sellicks Hill, SA | - | - | - |  | 1.47 | 0.19, 12.40 | 1.000 |  | - | - | 0.474 ^c^ |  | - | - | 1.000 ^c^ |
| 30 | Strathalbyn, SA | - | - | - |  | 0.28 | 0.00, 4.35 | 0.582 |  | - | - | - |  | - | - | - |
| **HOGGETS** | |  |  |  |  |  |  |  |  |  |  |  |  |  |  |  |
| 1 | Kojonup, WA | - | - | 1.000 ^c^ |  | - | - | 0.474 ^c^ |  | 1.00 | 0.10, 10.33 | 1.000 |  | - | - | - |
| 2 | Kojonup, WA | - | - | - |  | - | - | - |  | 0.27 | 0.02, 2.47 | 0.350 |  | - | - | - |
| 5 | Korunye, SA | - | - | - |  | - | - | 0.474 ^c^ |  | - | - | - |  | - | - | - |
| 9 | Watervale, SA | - | - | 1.000 ^c^ |  | 1.00 | 0.06, 17.8 | 1.000 |  | - | - | - |  | - | - | - |
| 10 | Broomehill, WA | - | - | - |  | 0.18 | 0.00, 2.45 | 0.303 |  | - | - | - |  | - | - | - |
| 12 | Tarlee, SA | - | - | 0.476 ^c^ |  | 1.83 | 0.23, 17.7 | 0.659 |  | - | - | 0.476 ^c^ |  | - | - | - |
| 13 | Giffard West, VIC | 2.16 | 0.10,147.1 | 1.000 |  | - | - | - |  | - | - | - |  | - | - | - |
| 15 | Katanning, WA | - | - | - |  | 0.16 | 0.01, 1.45 | 0.080 |  | - | - | - |  | - | - | - |
| 26 | Culla, VIC | - | - | 1.000 ^c^ |  | 1.47 | 0.19, 12.40 | 1.000 |  | - | - | 1.000 ^c^ |  | - | - | - |
| 29 | Ballarat, VIC | - | - | - |  | 0.30 | 0.00, 2.46 | 0.370 |  | - | - | 1.000 ^c^ |  | - | - | 1.000 ^c^ |
|  |  |  |  |  |  |  |  |  |  |  |  |  |  |  |  |  |
| **OVERALL^d^** |  | **0.71** | **0.24, 1.93** | **0.506** |  | **0.52** | **0.32, 0.83** | **0.007** |  | **0.51** | **0.22, 1.14** | **0.108** |  | **0.48** | **0.02, 5.31** | **0.561** |

^a^ SA: South Australia, VIC : Victoria, WA : Western Australia

^b^ Odds ratio for failure to rear calculated for ewes with specified *C. fetus* titre compared to ewes with titre below specified threshold

^c^ Odds ratio not calculated due to empty cell

^d^ Overall odds ratio calculated using logistic regression (flock included as fixed effect)
